# Phosphatidic acid phosphatase 1 impairs SARS-CoV-2 replication by affecting the glycerophospholipid metabolism pathway

**DOI:** 10.7150/ijbs.73057

**Published:** 2022-07-11

**Authors:** Bingpeng Yan, Shuofeng Yuan, Jianli Cao, Kingchun Fung, Pok-Man Lai, Feifei Yin, Kong-Hung Sze, Zhenzhi Qin, Yubin Xie, Zi-Wei Ye, Terrence Tsz-Tai Yuen, Kenn Ka-Heng Chik, Jessica Oi-Ling Tsang, Zijiao Zou, Chris Chun-Yiu Chan, Cuiting Luo, Jian-Piao Cai, Kwok-Hung Chan, Tom Wai-Hing Chung, Anthony Raymond Tam, Hin Chu, Dong-Yan Jin, Ivan Fan-Ngai Hung, Kwok-Yung Yuen, Richard Yi-Tsun Kao, Jasper Fuk-Woo Chan

**Affiliations:** 1 State Key Laboratory of Emerging Infectious Diseases, Carol Yu Centre for Infection, Department of Microbiology, School of Clinical Medicine, Li Ka Shing Faculty of Medicine, The University of Hong Kong, Pokfulam, Hong Kong Special Administrative Region, China.; 2 Key Laboratory of Tropical Translational Medicine of Ministry of Education, Hainan Medical University, Haikou, Hainan, China.; 3 Academician Workstation of Hainan Province, Hainan Medical University, Haikou, Hainan, China.; 4 Hainan Medical University-The University of Hong Kong Joint Laboratory of Tropical Infectious Diseases, The University of Hong Kong, Pokfulam, Hong Kong Special Administrative Region, China.; 5 Department of Pathogen Biology, Hainan Medical University, Haikou, Hainan, China.; 6 Centre for Virology, Vaccinology and Therapeutics, Hong Kong Science and Technology Park, Hong Kong Special Administrative Region, China.; 7 Department of Microbiology, Queen Mary Hospital, Pokfulam, Hong Kong Special Administrative Region, China.; 8 Department of Medicine, Li Ka Shing Faculty of Medicine, The University of Hong Kong, Pokfulam, Hong Kong Special Administrative Region, China.; 9 Department of Infectious Disease and Microbiology, The University of Hong Kong-Shenzhen Hospital, Shenzhen, China.; 10 School of Biomedical Sciences, Li Ka Shing Faculty of Medicine, The University of Hong Kong, Pokfulam, Hong Kong Special Administrative Region, China.; 11 Guangzhou Laboratory, Guangdong Province, China.

**Keywords:** COVID-19, lipidomics, phosphatidic acid phosphatases, SARS-CoV-2

## Abstract

Viruses exploit the host lipid metabolism machinery to achieve efficient replication. We herein characterize the lipids profile reprogramming *in vitro* and *in vivo* using liquid chromatography-mass spectrometry-based untargeted lipidomics. The lipidome of SARS-CoV-2-infected Caco-2 cells was markedly different from that of mock-infected samples, with most of the changes involving downregulation of ceramides. In COVID-19 patients' plasma samples, a total of 54 lipids belonging to 12 lipid classes that were significantly perturbed compared to non-infected control subjects' plasma samples were identified. Among these 12 lipid classes, ether-linked phosphatidylcholines, ether-linked phosphatidylethanolamines, phosphatidylcholines, and ceramides were the four most perturbed. Pathway analysis revealed that the glycerophospholipid, sphingolipid, and ether lipid metabolisms pathway were the most significantly perturbed host pathways. Phosphatidic acid phosphatases (PAP) were involved in all three pathways and PAP-1 deficiency significantly suppressed SARS-CoV-2 replication. siRNA knockdown of LPIN2 and LPIN3 resulted in significant reduction of SARS-CoV-2 load. In summary, these findings characterized the host lipidomic changes upon SARS-CoV-2 infection and identified PAP-1 as a potential target for intervention for COVID-19.

## Introduction

Coronaviruses are enveloped, positive-sense, single-stranded RNA viruses that can infect a wide range of human and animal species 1, 2. Since its first identification in late 2019, severe acute respiratory syndrome coronavirus 2 (SARS-CoV-2) has caused more than 455 million cases of Coronavirus Disease 2019 (COVID-19) including over 6 million deaths, and significant socioeconomic disruptions worldwide 3-5. With SARS-CoV-2's continuous adaptations in human, variants with altered pathogenicity and/or transmissibility have appeared 6, 7. The most recently emerged Omicron (B.1.1.529) variant is characterized by an unusually high number of amino acid mutations at its spike protein which renders it to be less susceptible to the neutralizing antibody response elicited by previous COVID-19 vaccines 8, 9. Moreover, it is highly transmissible despite having lower pathogenicity 10, 11. This rapidly spreading Omicron variant has caused resurgence of COVID-19 cases and major healthcare burdens in many parts of the world 12-16.

Virus-targeting antivirals such as monoclonal antibodies and enzyme inhibitors may become ineffective when resistant virus strains emerge 17. Thus, host-targeting antivirals against conserved host pathways or proteins that are less likely to become resistant may serve as important broad-spectrum treatment strategies for different variants of SARS-CoV-2 as well as different coronaviruses. Viruses are obligate intracellular parasites that rely on the host metabolism machinery for efficient replication 18. Lipids are involved in multiple stages of the life cycle of viruses, including coronaviruses 19-22. Lipidomics facilitates the identification of druggable host targets for different viruses, including coronaviruses 23-26. In this study, we investigated the host lipidomic perturbations induced by SARS-CoV-2 infection *in vitro* and *in vivo*. Pathway analysis and downstream validation studies identified phosphatidic acid phosphatase (PAP) as essential host factors for efficient SARS-CoV-2 replication and may be a potential treatment target for COVID-19.

## Materials and Methods

### Ethical approvals

The use of clinical specimens was approved by the Institutional Review Board of the University of Hong Kong / Hospital Authority Hong Kong West Cluster. Plasma samples were collected from COVID-19 patients whose respiratory tract specimens tested positive for SARS-CoV-2 and blood donors who tested negative for SARS-CoV-2 by quantitative reverse transcription-polymerase chain reaction (RT-qPCR).

### Viruses and cells

SARS-CoV-2 wild-type (HKU-001a strain; GenBank accession number MT230904) strain was isolated from respiratory tract specimens of laboratory-confirmed COVID-19 patients in Hong Kong 27, 28. The virus strains were propagated in VeroE6 cells and kept at -80°C in aliquots until use. Plaque assay was performed to titrate the cultured SARS-CoV-2. Briefly, HES2 cells were dissociated with Accutase (Invitrogen) into single cells suspension on day 0. Cells were seeded on low-attachment culture vessels (Corning) and cultured in mTeSR1 medium supplemented with 40µg/ml Matrigel, 1ng/ml BMP4 (Invitrogen) and 10µM Rho kinase inhibitor (ROCK) (R&D) under hypoxic environment with 5% O_2_. From day 1 to 3, cells were cultured in StemPro34 SFM (Invitrogen) with 50µg/ml ascorbic acid (AA) (Sigma), 2mM Gluta-MAX (Invitrogen), 10ng/ml BMP4, and 10ng/ml human recombinant activin-A (Invitrogen). From day 4 to 7, 5µM Wnt inhibitor IWR-1, a Wnt inhibitor (Tocris) was added. From day 8 to 14, cells were cultured under normoxia in RPMI 1640 medium (Invitrogen) supplemented with 2mM Gluta-MAX, 1×B-27 supplement (Invitrogen) and 50µg/mL AA. The cells were then dissociated with Accutase and seeded as monolayer in desired culture vessels for 3 days before infections. Vero-E6 (ATCC® CRL-1586™), and human colonic (Caco-2, ATCC® HTB-37™) cells were maintained in Dulbecco's modified eagle medium (DMEM, Gibco, CA, USA) culture medium supplemented with 10% heat-inactivated fetal bovine serum (FBS, Gibco), 100U/mL penicillin, and 100μg/ml streptomycin. A549-hACE2-TMPRSS2 cells (Invivogen) were cultured according to the protocol provided by the manufacturer. All cell lines were confirmed to be free of mycoplasma contamination by PlasmoTest (InvivoGen).

### Plasma collection and lipid extraction

Lipid extraction was performed on the subjects' plasma samples for liquid chromatography-mass spectrometry (LC-MS) analysis as we previously described with slight modifications 24, 25, 29. Briefly, 50µL of plasma was added to 20µL ice-cold methanol containing internal standards and butylated hydroxytoluene (BHT). The samples were vortex for 5s and kept on ice during subsequent extraction steps. Then, 190µL of chloroform/methanol (v/v 14:5) was added, followed by vortex for 30s, leaving the samples on ice and vortex again for 30s. The samples were further shaken for 5min at 1500 rpm at 4°C in the orbital mixer. The samples were then centrifuged at 4500 rpm for 10min at 4°C. The bottom phase was transferred to glass vials and was split into two aliquots (75µL for negative and another 70µL for positive). Finally, all aliquot samples were dried in a Labconco Centrivap cold trap concentrator for storage at -80°C until use.

### Coronavirus-infected cell-based lipid extraction

Caco-2 cells were maintained in Dulbecco's modified Eagle medium (DMEM) supplemented with 10% heat-inactivated fetal bovine serum (FBS), 100U/mL penicillin, and 100µg/mL streptomycin and incubated in 5% CO_2_ at 37°C. Archived clinical isolates of SARS-CoV-1 and SARS-CoV-2 isolated from patients were available at the Department of Microbiology, The University of Hong Kong. The viruses were cultured in Caco-2 cells in serum-free DMEM supplemented with 100 U/mL penicillin and 100µg/mL streptomycin. Cell-based lipid extraction and inactivation of virus infectivity were according to a previously described protocol 24-26. Briefly, cells were dissociated by using 550µL of ice-cold 150mM ammonium bicarbonate and 50µL of cell suspension was applied to perform DNA extraction for normalization 30. The remaining 500µL cell suspension was added 250µL of methanol containing internal standards and BHT. Then, 2mL of chloroform/methanol (v/v 3:1) was added, followed by vortexing and centrifugation at 4500rpm for 10min at 4°C. The bottom phase was transferred to glass vials and dried in a Labconco Centrivap cold trap concentrator for storage at -80°C.

### Liquid Chromatography-Mass Spectrometry (LC-MS)-based untargeted lipidomics

Untargeted lipidomics analysis was performed as previously described 24-26. Briefly, the lipid extract were reconstituted in 25µL of chloroform-methanol (1:1, v/v) and diluted to 1:10 of the original concentration of cell lysate in 225µL IPA-ACN-water (2:1:1, v/v/v) 31. The lipid samples were analyzed using an Acquity UPLC system coupled to a Synapt G2-Si High Definition Mass Spectrometry (HDMS) system (Waters Corp., Milford, MA, USA). The chromatography was performed on a Waters ACQUITY CSH C18 column (100mm × 2.1mm; 1.7µm) coupled to an Acquity CSH C18 VanGuard pre-column (5mm × 2.1mm; 1.7µm) (Waters; Milford, MA, USA). The column and autosampler temperature were maintained at 55^o^C and 4^o^C, respectively. The injection volume was 7µL for negative and 5µL for positive. The applied chromatography gradients and mobile phase composition were described in **Table S1**. The mass spectrometer was operated in MS^E^ mode and the data were acquired in both positive and negative modes. The capillary voltage was maintained at 2.5kV (positive mode) and 2.0kV (negative mode). Mass spectral data were acquired over the m/z range of 100 to 1500. MS and MS/MS acquisition were operated in the same parameters. Collision energy was applied at the range from 30 to 45eV for obtaining fragmentation pattern that provide identification and structural elucidation of the significant lipids. A total of 14 lipid internal standards were applied for sample preparation and LC-MS analysis for monitoring the lipids coverage and extraction efficiency including of Arachidonic acid-d8, Platelet-activating factor C-16-d4 (PAF C-16-d4), PE (17:0/17:0), PG (17:0/17:0), PC(17:0/0:0), C17 Sphingosine, C17 Ceramide, SM (d18:1/17:0), PC (12:0/13:0), Cholesterol (d7), TG (17:0/17:1/17:0) d5, DG (12:0/12:0/0:0), DG (18:1/2:0/0:0), PE (17:1/0:0) and commercial standards used for lipids identification. They all were purchased from Cayman Chemical (Ann Arbor, MI, USA) and Avanti Polar Lipids, Inc (Alabaster, AL, USA). Moreover, to check the stability of the LC-MS system during the analytical batch, QC samples were injected at the beginning of the run and after every 6 or 8 samples for monitoring the system variation. QC samples were pooled and prepared by mixing equal aliquots of all the biological samples 32-34.

### Data processing, statistical analysis, and lipids identification

All lipidomics data was processed to a usable data matrix by the MS-DIAL software for further analysis 35, 36. MetaboAnalyst 5.0 (http://www.metaboanalyst.ca) and SIMCA-P V12.0 (Umetrics, Umeå, Sweden) were used for univariate and multivariate analyses, respectively. Prior to statistical analysis, the data matrix needs to perform QC or DNA-based normalization for better comparison 35-37. The significant lipid features were identified by matching accurate MS and MS/MS fragmentation pattern data from the public database such as the MS-DIAL internal lipid database 35, MassBank of North America (MoNA, http://mona.fiehnlab.ucdavis.edu/), METLIN database (http://metlin.scripps.edu/), and LIPID MAPS (http://www.lipidmaps.org/). For confirmation of lipid identity using authentic chemical standards, the MS/MS fragmentation patterns of the chemical standards were compared with those of the candidate lipids measured under the same LC-MS condition. MetaboAnalyst and KEGG mapper were used to perform significant lipids pathway analyses 38.

### Gene silencing and overexpression assay

Caco-2 cells and A549-hACE2-TMPRSS2 cells in 24-well plates containing about 10^5^ cells per well were transfected with a 500µL mixture containing 60nM siRNA, 2µL Lipofectamine RNAiMAX (Thermo Fisher), Opti-MEM (Invitrogen), and antibiotic-free cell culture medium. At 24h after third transfection, the medium was replaced, and the cells were infected with SARS-CoV-2 (MOI=0.10). At 24h or 48h post-infection (hpi), the cells were collected into RLT lysis buffer (Qiagen, Hilden, Germany), followed by total nucleic acid extraction. The viral RNA load was determined by quantitative reverse transcription-polymerase chain reaction (qRT-PCR). The values obtained using scrambled siRNA-transfected cells were set as negative controls. For gene overexpression assay, LIPIN3 plasmids (Origene, RC215286) were transfected using Lipofectamine 3000 reagent (Thermo Fisher) into Caco-2 cell in 24-wells plate and collected the cell lysate samples in 48hpi (MOI=0.10), followed by nucleic acid extraction. The viral RNA load was determined by quantitative reverse transcription-polymerase chain reaction (qRT-PCR). The values obtained using mock-transfected cells were set as negative controls.

### Statistical analysis

Normality check was performed for the lipid features of each study group using Shapiro-Wilk test. False discovery rate (FDR) multiple test correction was performed for univariate analysis 39. For Caco-2 cell samples, FDR adjust P<0.05 and fold change >1.25 or <0.8 were used as the cut-off value. For human plasma samples, the FDR adjust P<0.05 and fold change >1.5 or <0.67 were used as the criteria for significant features selection. In multivariate analysis, the lipid features were first subjected to Pareto scaling and then followed by partial least squares discriminant analysis (PLS-DA) to find important variables with discriminative power. PLS-DA model was evaluated with the relevant R2 and Q2. The Variable Importance in Projection (VIP), which reflects both the loading weights for each component and the variability of the response explained by this component, was used to select the features 40. For virologicla analyses, statistical differences were calculated using two-tailed unpaired Student's t-test or one-way analysis of variance (ANOVA) where appropriateectively with GraphPad Prism 7. Differences were considered statistically significant when P<0.05.

## Results

### Lipidomic perturbations in SARS-CoV-2 infection *in vitro*

**Figure 1:**
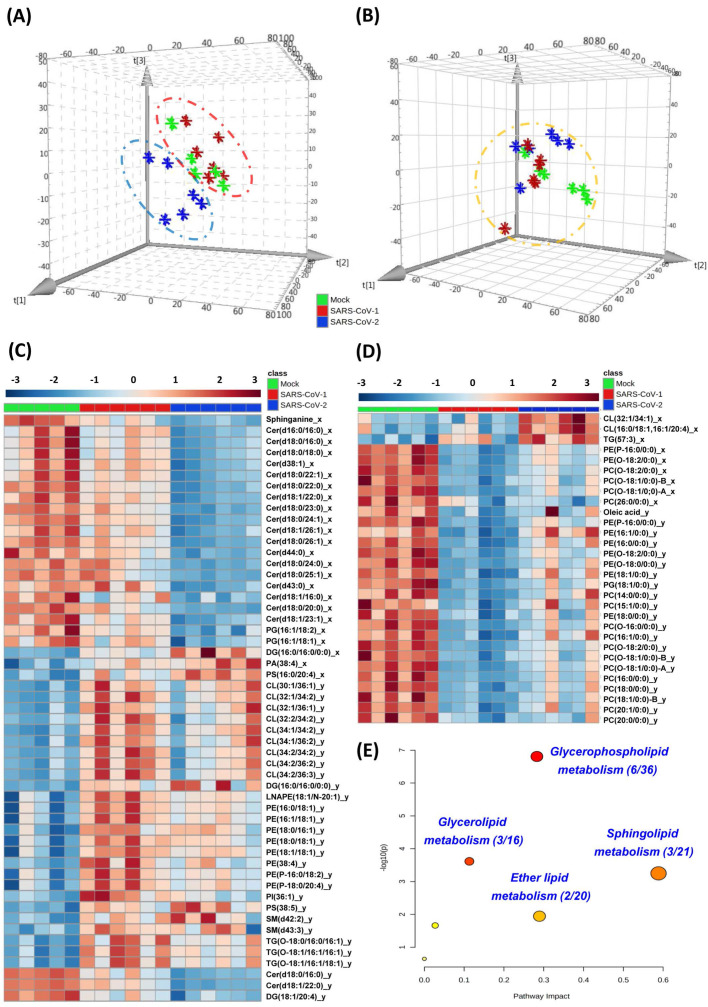
** Cell-based lipidomics of SARS-CoV-2 and SARS-CoV-1 infections*.*** (A) and (B) Principal component analysis (PCA) score plots showing the pattern of distribution of lipids at (A) 8 hours post-infection (hpi) and (B) 24 hpi (B) in SARS-CoV-2-, SARS-CoV-1-, or mock-infected Caco-2 cells (6 samples per group). The “⋇” symbols represent the individual cell samples. (C) and (D) Hierarchical clustering analysis was generated based on all significantly changed lipids between SARS-CoV-2-infected or SARS-CoV-1-infected and mock-infected samples at (C) 8 hpi and (D) 24 hpi. The “_x”-labelled and “_y”-labeled lipids represent significantly changed lipids between SARS-CoV-2-infected (“_x”-labelled) or SARS-CoV-1-infected (“_y”-labelled) and mock-infected, respectively. Each bar represents an identified lipid colored by its average intensity on a normalized scale from blue (decreased) to red (increased). (E) Pathway analysis of SARS-CoV-2-infected Caco-2 cells lipidomic changes was carried out by MetaboAnalyst. The x-axis represents the value calculated from the pathway topology analysis. The y-axis represents the transformation of the original p-value calculated from the enrichment analysis.

Lipidomics analysis was performed in human colorectal Caco-2 cells infected with either SARS-CoV-2 or SARS-CoV-1 (quality control data were shown in **Table S2**). Principal component analysis (PCA) score plots were used to illustrate the cell lipidome upon viral or mock infection at different time points. The lipidome of SARS-CoV-2-infected samples was markedly different from that of SARS-CoV-1-infected or mock-infected samples at 8hpi (**Figure 1A**) and more similar at 24hpi (**Figure 1B**). Corroboratively, the heatmap demonstrated more differentially perturbed lipids between SARS-CoV-2-infected samples and SARS-CoV-1-infected or mock-infected samples at 8hpi (**Figure 1C**) than at 24hpi (**Figure 1D**). At 8hpi, most of the changes in SARS-CoV-2 infection were ceramides with downregulation, whereas upregulation of multiple lipids, such as cardiolipins (CL) and phosphatidylethanolamines (PE), were observed in SARS-CoV-1 infection (**Tables S3 and S4**). Interestingly, at 8hpi, we showed that around 90% (17/20) of the lipids that were significantly different between SARS-CoV-2 and SARS-CoV-1 were also downregulated ceramides that belong to the sphingolipid metabolism pathway (**Table S5**). Thus, the suppressed sphingolipid metabolism was a hallmark to distinguish SARS-CoV-1 or SARS-CoV-2 at the early stage of infection. At 24hpi, most of the ether lipids were downregulated in SARS-CoV-2 and SARS-CoV-1 infections. Pathway analysis revealed that glycerophospholipid metabolism (hit rate, 6 hit lipids / total 36 lipids in this pathway), glycerolipid metabolism (hit rate, 3/16), sphingolipid metabolism (hit rate, 3/21) and ether lipid metabolism (hit rate, 2/20) were the four most significantly perturbed pathways after SARS-CoV-2 infection (**Figure 1E**).

### *In vivo* lipidomic changes in COVID-19 patients' plasma samples

**Figure 2:**
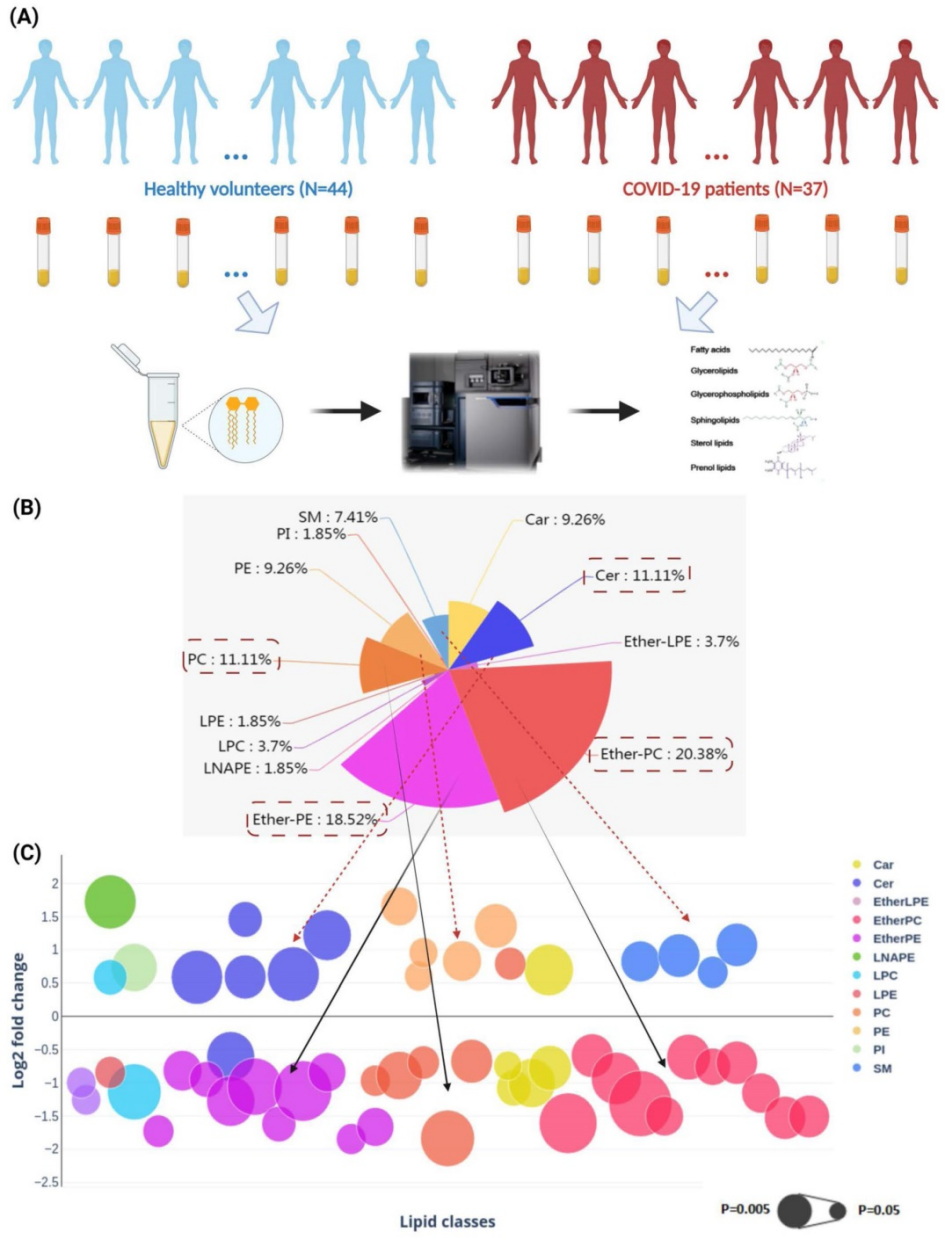
** Perturbed lipids in COVID-19 patients' plasam samples*.*** (A) The schematic of liquid chromatography-mass spectrometry-based lipidomics of COVID-19 patients' and non-infected control subjects' plasma samples. (B) Pie chart and (C) bubble plot showing 12 significantly perturbed lipid classes in COVID-19 patients' plasma samples. The x-axis and the y-axis in the bubble plot reprsent the lipid classes and log2-fold change in the plasma samples of COVID-19 patients compared to those of non-infected control subjects. The size of the bubbles represent the statistical significance of the change (FDR adjusted p-value from multiple t-test with the bigger bubble size representing a smaller p-value). Car, carnitine; Cer, ceramides; EtherLPE, ether-linked lysophosphatidylethanolamine; EtherPC, ether-linked phosphatidylcholine; EtherPE, ether-linked phosphatidylethanolamine; LNAPE, n-acyl-lysophosphatidyl-ethanolamine; LPC, lysophosphatidylcholine; LPE, lysophosphatidylethanolamine; PC, glycerophosphocholines; PE, glycerophosphoethanolamines; PI, glycerophosphoinositols; SM, sphingomyelin.

To characterize the lipid profile changes after SARS-CoV-2 infection in human and to validate the pathway perturbations identified in our cell culture model, we conducted LC-MS-based untargeted lipidomics analysis on the plasma samples of 37 COVID-19 patients and 44 non-infected controls (**Figure 2A**). There were 20 male and 17 female COVID-19 patients and 24 male and 20 female non-infected control subjects. The mean age was 55.4 and 57.5 years for the COVID-19 patients and non-infected control subjects, respectively. Our analysis detected a total of 622 known lipid features in positive mode and 311 known lipid features in negative mode. The coefficient of variation (CV) of all spike standards in all subjects and the quality controls (QC) samples were lower than 25% and 10%, respectively (**Table S6**) 33. After proceeding to statistical analysis and structural confirmation, a total of 54 significantly changed lipids were finally identified (**Table S7**).

These lipids belonged to 12 lipid classes, including carnitine (Car), ceramides (Cer), ether-linked lysophosphatidylethanolamine (EtherLPE), ether-linked phosphatidylcholine (EtherPC), ether-linked phosphatidylethanolamine (EtherPE), n-acyl-lysophosphatidyl-ethanolamine (LNAPE), lysophosphatidylcholine (LPC), lysophosphatidylethanolamine (LPE), phosphatidylcholine (PC), phosphatidylethanolamines (PE), phosphatidylinositols (PI), and sphingomyelin (SM) (**Figure 2B**) 41. Among them, EtherPC (11/54, 20.38%), EtherPE (10/54, 18.52%), PC (6/54, 11.11%), and Cer (6/54, 11.11%) were the four most perturbed lipid classes in COVID-19 patients' plasma samples. We then compared the direction and magnitude of these perturbed lipids (**Figure 2C**). Around two-thirds (35/54, 64.81%) of the perturbed lipids, including mainly Ether PC, Ether PE, and PCs, were downregulated, and the other one-third (19/54, 35.19%) of the perturbed lipids, including five Cers, five PEs, and four SMs, were significantly upregulated (**Figure 2C**).

### Perturbed pathway and receiver operating characteristic (ROC) curve analyses

**Figure 3:**
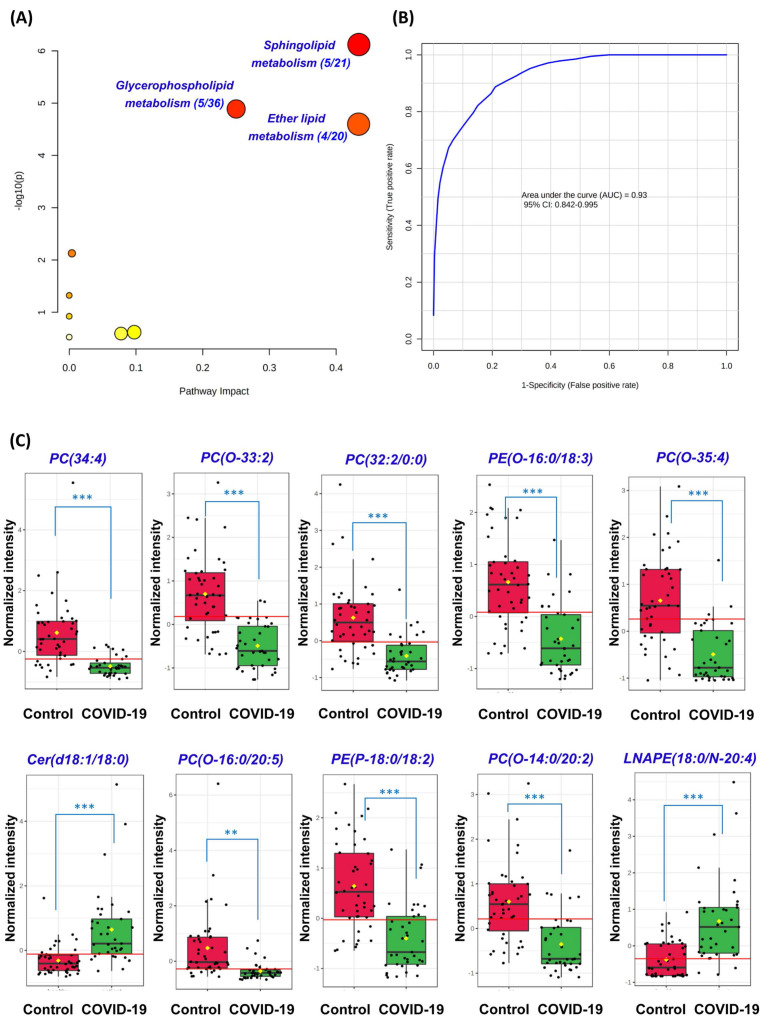
** Perturbed pathway and receiver operating characteristic (ROC) curve analyses*.*** (A) Pathway analysis of the lipidomic changes in COVID-19 patients' plasma samples using MetaboAnalyst. The x-axis represents the value calculated from the pathway topology analysis. The y-axis represents the transformation of the original p-value calculated from the enrichment analysis. (B) Ten lipids were selected by area under receiver operating characteristic (AUROC) >0.8. The figure was generated using MetaboAnalyst with ten lipids combination model. Feature ranking method was chosen as univariate AUROC. (C) Boxplots showing the different normalized intensitieis of the ten lipids between the COVID-19 patients' and non-infected control subjects' plasma samples. The horizontal red lines indicate the optimized cutoff values. The yellow diamonds and black hortizontal lines indicate the mean and median concentrations of each group, respectively. The “O-” prefix indicates the presence of an alkyl ether substituent, e.g., PC (O-16:0/20:5) and the “P-” prefix indicates the 1Z-alkenyl ether (Plasmalogen) substituent, e.g., PE (P-18:0/18:2). **, P<0.01; ***, P<0.001 (Student's t-test).

Next, we conducted pathway analysis which revealed sphingolipid metabolism (hit rate, 5/21), glycerophospholipid metabolism (hit rate, 5/36), and ether lipid metabolism (hit rate, 4/20) to be the three dominant perturbed pathways in COVID-19 patients' plasma samples (**Figure 3A**). All 54 significantly perturbed lipids (**Table S7**) were used to perform ROC curve analysis and the top 10 lipids ranked by area under ROC curve (AUROC) were selected to generate the ROC curve plot (**Figure 3B**). The 10 lipids consisted of 4 Ether PCs, 2 Ether PEs, 2 PCs, 1 Cer, and 1 LNAPE (**Figure 3C**). The ROC curve plot based on the top 10 lipids exhibited excellent discrimination between the plasma samples of COVID-19 patients and healthy controls. Corroborative with our pathway analysis results, these lipids also belonged to the glycerophospholipid, sphingolipid, and ether lipid classes. Collectively, these findings showed that glycerophospholipid, sphingolipid, and ether lipid metabolisms were markedly perturbed in both cell culture model and COVID-19 patients' plasma samples.

### Inhibition of PAP-1 enzyme significantly reduces SARS-CoV-2 replication

**Figure 4:**
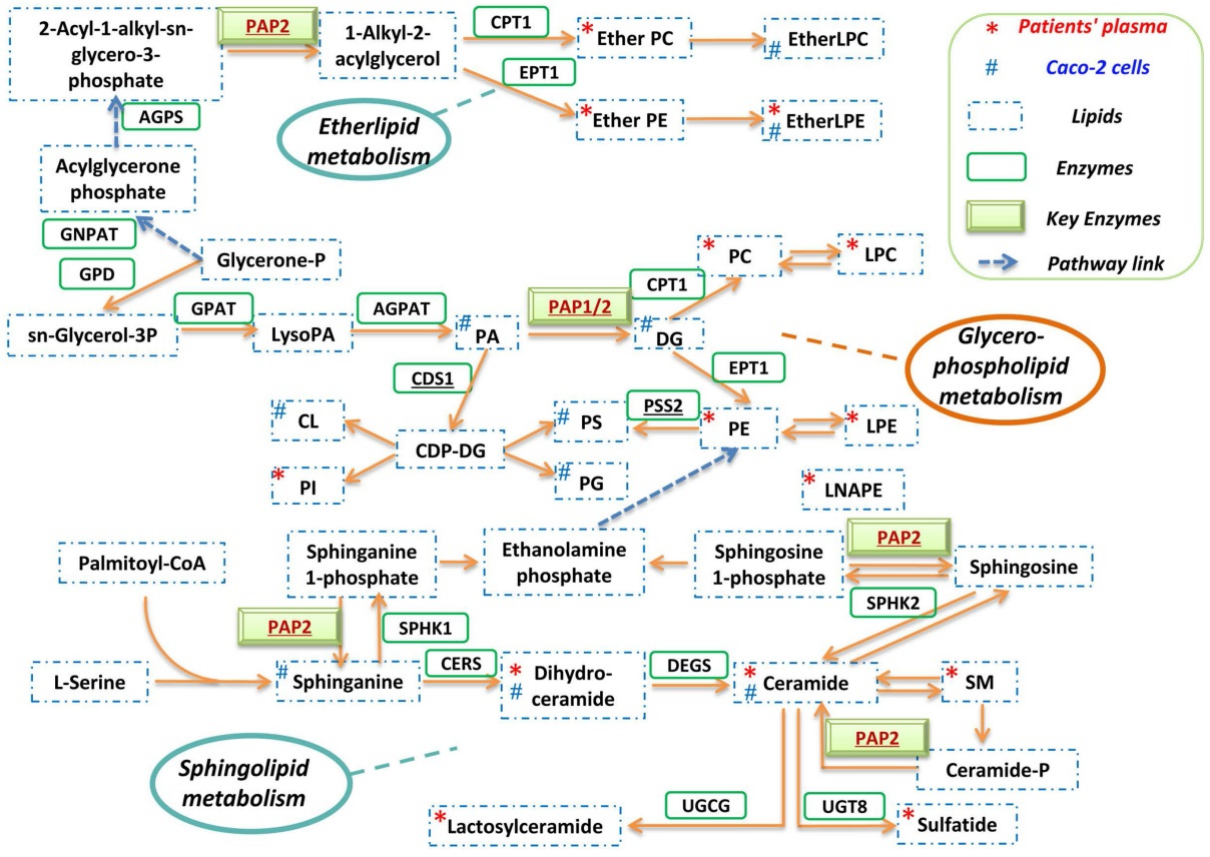
** Reprogrammed lipid pathways in SARS-CoV-2 infection*.*** The pathway map was manually constructed based on the significantly changed lipids in the in vitro Caco-2 cell-based and in vivo human plasma-based lipidomics studies with reference to the KEGG PATHWAY Database.

**Figure 5:**
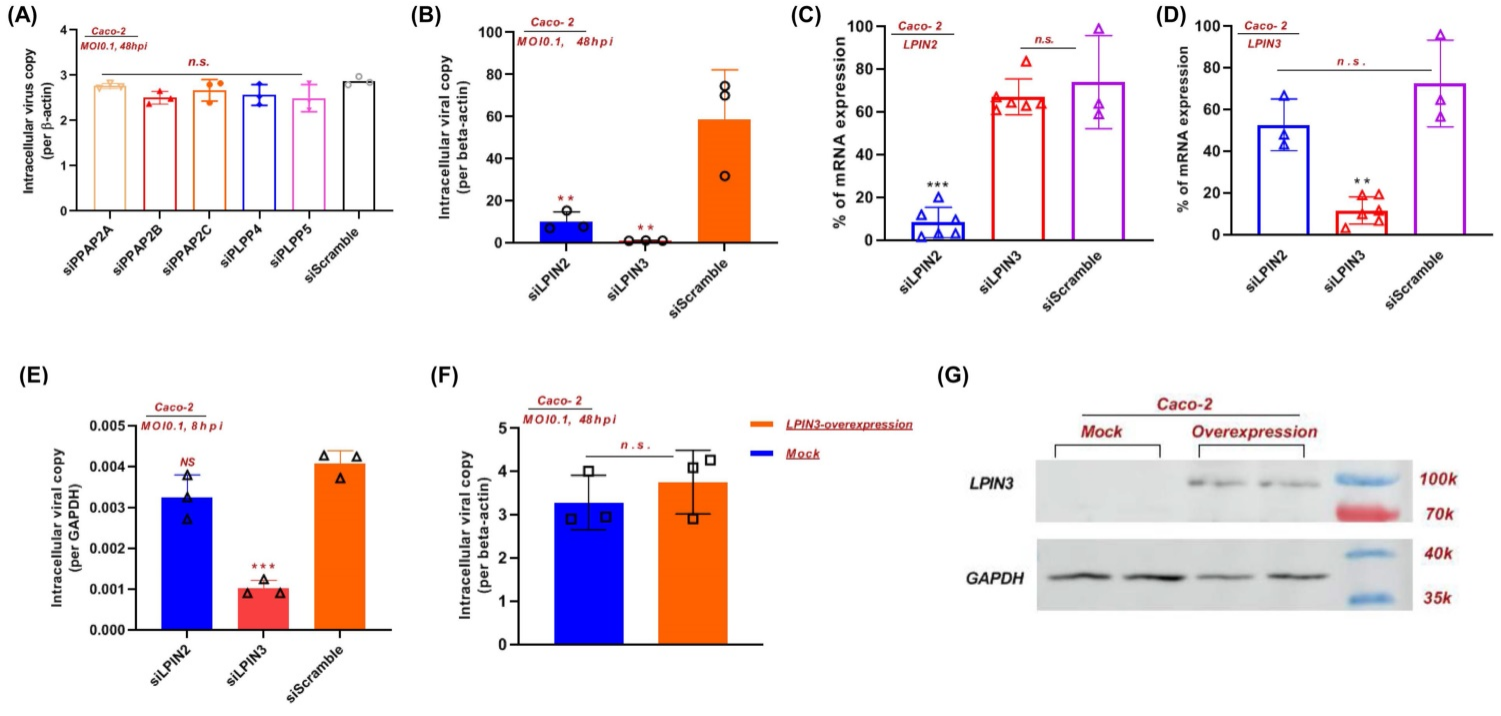
** PAP-1 is essential for efficent SARS-CoV-2 replication*.*** (A) siRNA knockdown of PP2A, PP2B, PP2C, PLPP4, and PLPP5, and (B) LPIN2 and LPIN3 were performed in Caco-2 cells before SARS-CoV-2 infection for 48 hours (multiplicity of infection, MOI = 0.1). The viral yields in the cell lysates were determined by RT-qPCR and normalized with human β-actin. (C and D) The individual siRNA knockdown efficiency and specificity of (C) LPIN2 and (D) LPIN3. Results are shown as % of scramble siRNA-treated. **, P<0.01; ***, P<0.001 (Student's t-test). (E) siRNA knockdown of LPIN2 and LPIN3 were performed in Caco-2 cells before SARS-CoV-2 infection for 8 hours (MOI = 0.1). (F) LPIN3 overexpression plasmid was transfected to Caco-2 cells before SARS-CoV-2 infection for 48 hours (MOI = 0.1). (G) Western blot showing the overexpression of LPIN3 protein.

A globally reprogrammed lipids pathway map based on the identified perturbed pathways was constructed (**Figure 4**). Among the three pathways, glycerophospholipid metabolism is the central pathway that links the other two pathways. In the glycerophospholipid pathway, glycerone-P is firstly metabolized to *sn*-Glycerol 3-phosphate, then further metabolized to lysoPA, and finally converted to PA, DG, and other glycerophospholipids. Importantly, PAP-1/2 is involved in all three pathways. We therefore further investigated the effects of PAP-1/2 on SARS-CoV-2 infection. To determine if PAP-1 and/or PAP-2 affect SARS-CoV-2 replication, siRNA knockdown and gene overexpression assays were applied to SARS-CoV-2-infected Caco-2 cells. No significant change in viral genome copies was observed when the PAP-2-encoding genes (PPAP2A, PPAP2B, and PPAP2C), as well as other phosphatidic acid phosphatases-coding genes (PLPP4 and PLPP5) were individually knockdown (**Figure 5A**). Next, we investigated the PAP-1-encoding genes LPIN2 and LPIN3, as LPIN1 is poorly expressed in Caco-2 cells 42. In a multi-cycle virus growth experiment, significant reduction of viral genome copies in the lysates of siLPIN2-treated (P<0.01) or siLPIN3-treated (P<0.001) cells was evident, with higher dependence of SARS-CoV-2 on LPIN3 than LPIN2 (**Figure 5B**) despite similar knockdown efficiency (**Figure 5C and 5D**). In addition, siRNAs directed against LPIN2 or LPIN3 were introduced into virus-permissive human lung epithelial cells (A549-hACE2-TMPRSS2 cells) with favorable knockdown efficiency to validate the efficacy of PAP-1 inhibition (**Figure S1A and S1B**). Similarly, the viral genome copies in the lysates of siLPIN2-treated (P<0.01) or siLPIN3-treated (P<0.01) cells were remarkably decreased, and also higher dependence of SARS-CoV-2 on LPIN3 than LPIN2 (**Figure S1C**). In a single cycle virus growth experiment, siLPIN3 but not siLPIN2 significantly decreased intracellular viral genome copies (P<0.001) (**Figure 5E**). LPIN3-overexpression assay demonstrated no significant effect on SARS-CoV-2 replication (**Figure 5F and 5G**). Collectively, these results indicated that the PAP-1 enzyme is important for the efficient replication of SARS-CoV-2.

## Discussion

Many viruses including coronaviruses induce lipid metabolism remodeling in host cells to facilitate their own replication 26. The understanding on the major pathways and enzymes involved in virus-induced lipid metabolism remodeling may provide novel insights into the pathogenesis and potential treatment targets for COVID-19. In this study, we investigated the host lipidomics perturbations induced by SARS-CoV-2 infection *in vitro* and *in vivo*. We identified glycerophospholipid metabolism, sphingolipid metabolism, and ether lipid metabolism to be the dominantly perturbed pathways. Glycerophospholipid metabolism is the central pathway that links the sphingolipid and ether lipid metabolism pathways. Recent studies demonstrated marked differences between the glycerophospholipid composition of purified influenza virions and that of uninfected host cells, which may facilitate viral processing during infection 43. RNA viruses may subvert phosphatidylethanolamine (PE) to build membrane-bound viral replicase complexes for robust replication in PE-enriched membrane microdomains 22. Our previous study showed that the glycerophospholipid metabolism pathway was dramatically modulated by MERS-CoV infection and glycerophospholipid homeostasis played a critical role in optimizing coronavirus replication 26. The findings in the current study suggest that glycerophospholipid metabolism reprogramming may be a conserved strategy utilized by different coronaviruses for efficient replication. Corroboratively, a recent study demonstrated that SARS-CoV-2 replication is significantly reduced in cells which are deficient in the expression of AGPAT, another key enzyme involved in the glycerophospholipid metabolism pathway 44.

PAP-1 has important roles in the mediation of lipid metabolism that affects virus replication. As shown in figure 4, in the glycerophospholipid metabolism pathway, PAP-1 is substrate-specific only to phosphatidate (PA) and converts PA into diacylglycerol (DG). DG is a class of important lipid mediators that are converted into multiple glycerolipids along this pathway (e.g., TG, PC and PE). TG serves as an important cellular energy source and is lipolytically broken down into fatty acids (FAs), which are then imported into the mitochondria and consumed by β-oxidation to produce ATP 45. TG production thus provides the metabolic energy required for viral genome and protein production during the virus replication cycle. Moreover, as we previously reported, TG is also involved in lipid droplets (LDs) formation that served as anchors for viral proteins (e.g., SARS-CoV-2 nucleocapsid protein and spike protein) 46. PC and PE are major building blocks of membrane-bound viral replicase complexes for robust replication and envelope formation of various RNA viruses 22. Collectively, PAP-1 supports virus replication by regulating downstream lipid production that further affects multiple stages of the virus replication cycle, including metabolic energy supply, viral anchors, and replicase complex formation.

PAP-1/2 is involved in all three lipid metabolism pathways dominantly perturbed in SARS-CoV-2 infection. Mammalian cells contain two different isotypes of PAP. PAP-1 but not PAP-2 is Mg^2+^-dependent 47. Using siRNA knockdown and gene overexpression assays, we further showed that PAP-1 significantly affects SARS-CoV-2 replication and may be a potential host-based treatment target for COVID-19. PAP-1 is encoded by a group of genes named LPINs which are substrate-specific to only phosphatidate and is involved in glycerophospholipid, glycerolipid, and mTOR signaling pathways. The lipin family has three lipin proteins (lipin 1, lipin 2, and lipin 3), with each of them having PAP activity but distinct tissue distributions 42. The contribution of lipin 1, lipin 2, and lipin 3 to PAP activity in metabolic tissues are discriminant 48. In heart and skeletal muscle cells, lipin 1 is the predominant type, whereas lipin1 and lipin 2 demonstrate additive PAP activity in lung and brain cells. Lipin 1 is the predominant PAP enzyme in many tissues with the notable exception of the intestine which exclusively expresses lipin 2 and lipin 3. Our findings indicated that the deficiency of lipin 2 or/and lipin 3 could remarkably inhibit SARS-CoV-2 replication.

In summary, our findings identified PAP-1 as a key regulatory enzyme of the glycerophospholipid remodeling in SARS-CoV-2 infection. PAP-1 and/or lipin 3-targeting drug compounds may be a potential host-based treatment strategy for COVID-19.

## Supplementary Material

Supplementary figure and tables.
