# Supplementary material for: Phosphatidic acid phosphatase 1 impairs SARS-CoV-2 replication by affecting the glycerophospholipid metabolism pathway
Source: Int J Biol Sci. 2022 Jul 11;18(12):4744–55. doi: 10.7150/ijbs.73057 (PMC9305268; doi:10.7150/ijbs.73057)
Supplement: Supplementary file 1 — Supplementary figure and tables. [file ijbsv18p4744s1.pdf]

Supplementary information

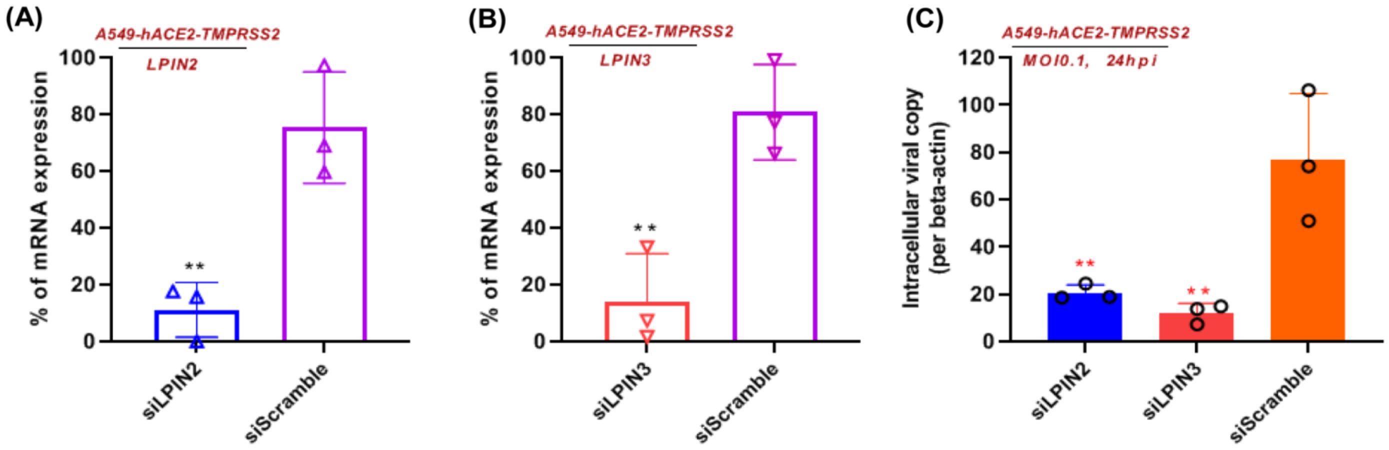

Figure S1. siRNA knockdown of LPIN2 and LPIN3 were performed in A549-hACE2-TMPRSS2 cells before SARS-CoV-2 infection for 24 hours (multiplicity of infection, MOI = 0.1). The individual siRNA knockdown efficiency of (A) LPIN2 and (B) LPIN3. Results are shown as % of scramble siRNA-treated. The viral yields in the cell lysates were determined by RT-qPCR and normalized with human β-actin. (C). For all statistical analysis above, **$p<0.01$ (Student's t-test).

Table S1. Gradient elution program applied for UPLC-MS analysis

Time (min)	Flow rate (mL/min)	Mobile phase A (%)	Mobile phase B (%)	Curve
Initial	0.4	60	40	6
2	0.4	57	43	6
2.1	0.4	50	50	6
12	0.4	46	54	6
12.1	0.4	30	70	6
18	0.4	1	99	6
18.1	0.4	60	40	6
20	0.4	60	40	6

The mobile phase A was 60:40 acetonitrile:water (v/v) with 10 mM ammonium formate and 0.1% formic acid. The mobile phase B for positive mode was 90:10 isopropanol:acetonitrile (v/v) with 10 mM ammonium formate and 0.1% formic acid. The mobile phase B for negative mode was 90:10 isopropanol:acetonitrile (v/v) with 10 mM ammonium acetate.

Table S2. List of the mixture of 14 internal standards with corresponding statistical information in cell based lipidomics study

Common Name	Formula	Detection mode	Adduct ion name	m/z	Retention time (min)	CV of cell samples	CV of QC samples
Ceramide C17	C35H69NO3	Positive	[M+H-H2O]+	534.5351	11.61	8.33%	11.03%
Ceramide C17	C35H69NO3	Positive	[M+Na]+	574.5294	11.62	8.76%	0.04%
Cholesterol d7	C27H39D7O	Positive	[M-H2O+H]+	376.4038	6.66	13.73%	0.13%
DG (12:0/12:0/0:0)	C27H52O5	Positive	[M+Na]+	479.3819	5.16	11.37%	9.54%
DG (12:0/12:0/0:0)	C27H52O5	Positive	[M+NH4]+	474.4257	5.19	11.93%	3.22%
DG (18:1/2:0/0:0)	C23H42O5	Positive	[M+Na]+	421.3032	2.87	16.62%	0.99%
DG (18:1/2:0/0:0)	C23H42O5	Positive	[M+NH4]+	416.3467	2.88	24.98%	6.67%
LPC (17:0)	C25H52NO7P	Positive	[M+H]+	510.3665	1.73	9.54%	0.64%
LPE (17:1)	C22H44NO7P	Positive	[M+H]+	466.3037	1.37	10.14%	0.07%
PAF C-16-d4	C26H50D4NO7P	Positive	[M+H]+	528.4086	1.87	6.59%	6.85%
PC (12:0/13:0)	C33H66NO8P	Positive	[M+H]+	636.4741	3.63	9.18%	8.89%
PE (17:0/17:0)	C39H78NO8P	Positive	[M+H]+	720.5711	13.04	9.60%	5.78%
SM (17:0)	C40H81N2O6P	Positive	[M+H]+	717.5905	8.13	22.68%	7.01%
Sphingosine (d17:1)	C17H35NO2	Positive	[M+H]+	286.2805	1.19	10.99%	0.05%
TG (17:0/17:1/17:0)-d5	C54H97D5O6	Positive	[M+Na]+	874.8035	16.09	23.66%	4.70%
TG (17:0/17:1/17:0)-d5	C54H97D5O6	Positive	[M+NH4]+	869.7181	16.09	23.83%	5.08%
Ceramide C17	C35H69NO3	Negative	[M+Cl]-	586.4970	12.64	18.78%	3.23%
Arachidonic acid-d8	C20H24D8O2	Negative	[M-H]-	311.2823	2.44	24.44%	0.58%
LPE (17:1)	C22H44NO7P	Negative	[M-H]-	464.2783	1.42	11.86%	2.74%
PE (17:0/17:0)	C39H78NO8P	Negative	[M-H]-	718.5383	13.24	22.85%	9.62%
PG (17:0/17:0)	C40H79O10P	Negative	[M-H]-	749.5338	11.13	7.23%	6.45%

Abbreviations: DG, diacylglycerol; LPC, lysophosphatidylcholine; LPE, lysophosphatidylethanolamine; PC, phosphatidylcholine; PE, phosphatidylethanolamine; PG, phosphatidylglycerol; SM, sphingomyelin; TG, triacylglycerol.

Table S3. Lipids that were significantly different between SARS-CoV-2-infected and mock-infected Caco-2 cells

Significant lipids	Detection mode	Retention time (min)	Accurate mass in detection mode	Adduct ion name	lipids species	Identification information	p-Value in 8h SARS-CoV-2 infected Cell vs Mock	Fold change in 8h SARS-CoV-2 infected Cell vs Mock	p-Value in 24h SARS-CoV-2 infected Cell vs Mock	Fold change in 24h SARS-CoV-2 infected Cell vs Mock
Sphinganine	positive	1.375	302.30566	[M+H]+	SPB	STD	0.0007	0.59	NS	NS
Cer(d18:0/23:0)	negative	14.534	682.63507	[M+HCOO]-	Cer	MS/MS	0.0001	0.48	NS	NS
Cer(d18:0/26:1)	negative	14.723	722.66699	[M+HCOO]-	Cer	MS/MS	0.0001	0.29	NS	NS
Cer(d18:0/25:1)	positive	14.287	664.66095	[M+H]+	Cer	MS/MS	0.0001	0.43	NS	NS
Cer(d18:1/16:0)	positive	9.173	538.52069	[M+H]+	Cer	STD	0.0185	0.55	NS	NS
Cer(d18:0/24:1)	negative	14.275	694.63568	[M+HCOO]-	Cer	MS/MS	0.0003	0.40	NS	NS
Cer(d18:0/22:0)	negative	14.312	668.61938	[M+HCOO]-	Cer	MS/MS	0.0004	0.39	NS	NS
Cer(d44:0)	negative	15.157	724.68146	[M+HCOO]-	Cer	MS/MS	0.0036	0.50	NS	NS
Cer(d18:1/22:0)	negative	14.037	680.61829	[M-H]-	Cer	MS/MS	0.0040	0.45	NS	NS
Cer(d43:0)	positive	13.629	666.6416	[M+NH4]+	Cer	MS/MS	0.0050	0.65	NS	NS
PS(16:0/20:4)	negative	5.722	782.49744	[M-H]-	PS	MS/MS	0.0050	1.60	NS	NS
Cer(d18:1/26:1)	negative	14.336	720.64746	[M+HCOO]-	Cer	MS/MS	0.0111	0.52	NS	NS
Cer(d18:0/22:1)	negative	13.784	666.60315	[M+HCOO]-	Cer	MS/MS	0.0158	0.37	NS	NS
Cer(d18:0/16:0)	negative	10.911	584.52637	[M+HCOO]-	Cer	STD	0.0326	0.34	NS	NS
PG(16:1/18:2)	negative	4.836	743.48297	[M-H]-	PG	MS/MS	0.0326	0.45	NS	NS
DG(16:0/16:0/0:0)	positive	13.259	591.49634	[M+Na]+	DG	MS/MS	0.0327	1.78	NS	NS
Cer(d38:1)	negative	13.187	638.56891	[M+HCOO]-	Cer	MS/MS	0.0370	0.48	NS	NS
PG(16:1/18:1)	positive	5.644	764.54572	[M+NH4]+	PG	MS/MS	0.0375	0.64	NS	NS
Cer(d16:0/16:0)	negative	8.093	556.49347	[M+HCOO]-	Cer	MS/MS	0.0393	0.39	NS	NS
PA(38:4)	negative	12.737	723.48206	[M-H]-	PA	MS/MS	0.0402	1.58	NS	NS
Cer(d18:0/18:0)	negative	13.176	612.55798	[M+HCOO]-	Cer	STD	0.0453	0.47	NS	NS
Cer(d18:0/20:0)	positive	13.6	596.59796	[M+H]+	Cer	MS/MS	0.0001	0.52	NS	NS
Cer(d18:1/23:1)	positive	13.659	634.61414	[M+H]+	Cer	MS/MS	0.0102	0.51	NS	NS
Cer(d18:0/24:0)	positive	14.544	652.66162	[M+H]+	Cer	MS/MS	0.0003	0.40	NS	NS
CL(16:0/18:1,16:1/20:4)	negative	15.411	1423.96094	[M-H]-	CL	MS/MS	NS	NS	0.0498	1.28
CL(32:1/34:1)	negative	15.427	1375.96179	[M-H]-	CL	MS/MS	NS	NS	0.0306	1.33
PC(O-18:1/0:0)-B	positive	2.183	508.37604	[M+H]+	EtherLPC	MS/MS	NS	NS	0.0469	0.71

PC(O-18:1/0;0)-A	positive	1.676	508.37607	[M+H]+	EtherLPC	MS/MS	NS	NS	0.0230	0.54
PC(O-18:2/0:0)	positive	1.617	506.3595	[M+H]+	EtherLPC	MS/MS	NS	NS	0.0077	0.56
PE(O-18:2/0:0)	negative	1.748	462.29834	[M-H]-	EtherLPC	MS/MS	NS	NS	0.0306	0.72
PE(P-16:0/0:0)	negative	1.675	436.28333	[M-H]-	EtherLPE	MS/MS	NS	NS	0.0306	0.75
TG(57:3)	positive	16.397	944.86566	[M+NH4]+	TG	MS/MS	NS	NS	0.0230	1.36

Remarks: MSMS = lipids that were confirmed with fragment pattern of database; STD = lipids that were confirmed with authentic standards; The 'O-' prefix is used to indicate the presence of an alkyl ether substituent, e.g., PC (O-16:0/0:0), whereas the 'P-' prefix is used for the 1Z-alkenyl ether (Plasmalogen) substituent, e.g., PE (P-16:0/16:0). Abbreviations: Cer, ceramide; CL, cardiolipin; DG, diacylglycerol; EtherLPC, ether-linked lysophosphatidylcholine; EtherLPE, ether-linked lysophosphatidylethanolamine; EtherPE, ether-linked phosphatidylethanolamine; LPC, lysophosphatidylcholine; PA, phosphatidate; PG, Phosphatidylglycerol; PS, phosphatidylserine; TG, triacylglycerol.

Table S4. Lipids that were significantly different between SARS-CoV-1-infected and mock-infected Caco-2 cells

Significant lipids	Detection mode	Retention time (min)	Accurate mass in detection mode	Adduct ion name	lipids species	Identification information	p-Value in 8h SARS-CoV-1 infected Cell vs Mock	Fold change in 8h SARS-CoV-1 infected Cell vs Mock	p-Value in 24h SARS-CoV-1 infected Cell vs Mock	Fold change in 24h SARS-CoV-1 infected Cell vs Mock
Cer(d18:0/16:0)	positive	10.15	540.5361	[M+H]+	Cer	STD	0.0376	0.64	NS	NS
Cer(d18:1/22:0)	positive	13.58	622.6140	[M+H]+	Cer	MS/MS	0.0409	0.72	NS	NS
CL(30:1/36:1)	positive	15.33	1395.0079	[M+NH4]+	CL	MS/MS	0.0427	1.35	NS	NS
CL(32:1/34:2)	positive	15.07	1392.9904	[M+NH4]+	CL	MS/MS	0.0347	1.27	NS	NS
CL(32:1/36:1)	positive	15.59	1423.0391	[M+NH4]+	CL	MS/MS	0.0432	1.40	NS	NS
CL(32:2/34:2)	positive	14.83	1390.9760	[M+NH4]+	CL	MS/MS	0.0436	1.26	NS	NS
CL(34:1/34:2)	positive	15.33	1421.0228	[M+NH4]+	CL	MS/MS	0.0283	1.30	NS	NS
CL(34:1/36:2)	positive	15.59	1449.0543	[M+NH4]+	CL	MS/MS	0.0398	1.33	NS	NS
CL(34:2/34:2)	positive	15.07	1419.0071	[M+NH4]+	CL	MS/MS	0.0336	1.26	NS	NS
CL(34:2/36:2)	positive	15.33	1447.0377	[M+NH4]+	CL	MS/MS	0.0353	1.27	NS	NS
CL(34:2/36:3)	positive	15.12	1445.0233	[M+NH4]+	CL	MS/MS	0.0356	1.26	NS	NS
DG(16:0/16:0/0:0)	positive	13.25	586.5421	[M+NH4]+	DG	MS/MS	0.0175	1.34	NS	NS
DG(18:1/20:4)	positive	12.18	660.5589	[M+NH4]+	DG	MS/MS	0.0353	0.78	NS	NS
LNAPE(18:1/N-20:1)	negative	9.90	770.5695	[M-H]-	LNAPE	MS/MS	0.0241	1.27	NS	NS
PE(16:0/18:1)	negative	10.48	716.5220	[M-H]-	PE	MS/MS	0.0241	1.26	NS	NS
PE(16:1/18:1)	negative	8.09	714.5078	[M-H]-	PE	MS/MS	0.0241	1.26	NS	NS
PE(18:0/16:1)	negative	7.26	716.5241	[M-H]-	PE	MS/MS	0.0241	1.32	NS	NS
PE(18:0/18:1)	negative	9.61	744.5544	[M-H]-	PE	MS/MS	0.0241	1.27	NS	NS
PE(18:1/18:1)	negative	7.46	742.5394	[M-H]-	PE	MS/MS	0.0241	1.30	NS	NS
PE(38:4)	positive	10.02	768.5557	[M+H]+	PE	MS/MS	0.0483	1.27	NS	NS
PE(P-16:0/18:2)	negative	9.19	698.5118	[M-H]-	EtherPE	MS/MS	0.0241	1.28	NS	NS
PE(P-18:0/20:4)	negative	9.31	748.5284	[M-H]-	EtherPE	MS/MS	0.0241	1.28	NS	NS
PI(36:1)	positive	8.74	882.6074	[M+NH4]+	PI	MS/MS	0.0398	1.27	NS	NS

PS(38:5)	positive	6.26	810.5299	[M+H]+	PS	MS/MS	0.0356	1.26	NS	NS
SM(d42:2)	positive	13.22	835.6670	[M+Na]+	SM	MS/MS	0.0429	1.26	NS	NS
SM(d43:3)	positive	13.00	825.6821	[M+H]+	SM	MS/MS	0.0283	1.30	NS	NS
TG(O-18:0/16:0/16:1)	positive	16.17	834.7936	[M+NH4]+	EtherTG	MS/MS	0.0358	1.26	NS	NS
TG(O-18:1/16:1/16:1)	positive	15.89	832.7758	[M+NH4]+	EtherTG	MS/MS	0.0364	1.25	NS	NS
TG(O-18:1/16:1/18:1)	positive	16.17	860.8087	[M+NH4]+	EtherTG	MS/MS	0.0365	1.27	NS	NS
Oleic acid	negative	3.07	281.2488	[M-H]-	FA	STD	NS	NS	0.0034	0.52
PC(14:0/0:0)	positive	1.03	468.3088	[M+H]+	LPC	MS/MS	NS	NS	0.0022	0.31
PC(15:1/0:0)	positive	1.51	480.3224	[M+H]+	LPC	MS/MS	NS	NS	0.0069	0.57
PC(16:0/0:0)	positive	1.38	518.3216	[M+Na]+	LPC	MS/MS	NS	NS	0.0008	0.13
PC(16:1/0:0)	positive	1.08	494.3257	[M+H]+	LPC	MS/MS	NS	NS	0.0024	0.50
PC(18:0/0:0)	positive	1.94	524.3718	[M+H]+	LPC	MS/MS	NS	NS	0.0007	0.48
PC(18:1/0:0)-B	positive	1.43	544.3383	[M+Na]+	LPC	MS/MS	NS	NS	0.0026	0.24
PC(20:0/0:0)	positive	2.81	552.4027	[M+H]+	LPC	MS/MS	NS	NS	0.0076	0.61
PC(20:1/0:0)	positive	2.00	550.3871	[M+H]+	LPC	MS/MS	NS	NS	0.0003	0.47
PC(O-16:0/0:0)	positive	1.61	482.3610	[M+H]+	EtherLPC	MS/MS	NS	NS	0.0003	0.32
PC(O-18:1/0:0)-A	positive	1.68	508.3761	[M+H]+	EtherLPC	MS/MS	NS	NS	0.0001	0.30
PC(O-18:1/0:0)-B	positive	2.18	508.3760	[M+H]+	EtherLPC	MS/MS	NS	NS	0.0012	0.60
PC(O-18:2/0:0)	positive	1.62	506.3595	[M+H]+	EtherLPC	MS/MS	NS	NS	0.0001	0.38
PE(16:0/0:0)	negative	1.47	452.2780	[M-H]-	LPE	MS/MS	NS	NS	0.0001	0.53
PE(16:1/0:0)	negative	1.12	450.2614	[M-H]-	LPE	MS/MS	NS	NS	0.0008	0.61
PE(18:0/0:0)	positive	2.02	482.3252	[M+H]+	LPE	MS/MS	NS	NS	0.0062	0.66
PE(18:1/0:0)	negative	1.54	478.2945	[M-H]-	LPE	MS/MS	NS	NS	0.0001	0.58
PE(O-18:0/0:0)	negative	1.64	466.3289	[M-H]-	EtherLPE	MS/MS	NS	NS	0.0001	0.29
PE(O-18:2/0:0)	negative	1.75	462.2983	[M-H]-	EtherLPE	MS/MS	NS	NS	0.0001	0.57
PE(P-16:0/0:0)	negative	1.68	436.2833	[M-H]-	EtherLPE	MS/MS	NS	NS	0.0001	0.60
PG(18:1/0:0)	negative	1.17	509.2872	[M-H]-	LPG	MS/MS	NS	NS	0.0002	0.55

Remarks: MSMS = lipids that were confirmed with fragment pattern of database; STD = lipids that were confirmed with authentic standards; The 'O-' prefix is used to indicate the presence of an alkyl ether substituent, e.g., PC (O-16:0/0:0), whereas the 'P-' prefix is used for the 1Z-alkenyl ether (Plasmalogen) substituent, e.g., PE (P-16:0/16:0). Abbreviations: Cer, ceramide; CL, cardiolipin; DG, diacylglycerol; EtherLPC, ether-linked lysophosphatidylcholine; EtherLPE, ether-linked lysophosphatidylethanolamine; EtherPC, ether-linked phosphatidylcholine; EtherPE, ether-linked phosphatidylethanolamine; LNAPE, n-acyl-lysophosphatidylethanolamine; LPC, lysophosphatidylcholine; PE, phosphatidylethanolamine; PG, Phosphatidylglycerol; PI, phosphatidylinositol; PS, phosphatidylserine; TG, triacylglycerol.

Table S5. Lipids that were significantly different between SARS-CoV-1 and SARS-CoV-2 infected cells

Lipids id	Significant lipids	Detection mode	Retention time	Accurate mass in detection mode	Adduct ion name	Lipids species	Identification information	p-Value in 8h SARS-CoV-2 infected Cell vs SARS-CoV-1	Fold change in 8h SARS-CoV-2 infected Cell vs SARS-CoV-1
495	Cer(d16:0/16:0)	negative	8.09	556.49347	[M+HCOO]-	Cer	MS/MS	0.0189	0.50
536	Cer(d18:0/16:0)	negative	10.91	584.52637	[M+HCOO]-	Cer	STD	0.0278	0.50
588	Cer(d18:0/18:0)	negative	13.18	612.55798	[M+HCOO]-	Cer	STD	0.0323	0.60
1393	Cer(d18:0/20:0)	negative	13.60	596.59796	[M+H]+	Cer	MS/MS	0.0269	0.66
704	Cer(d18:0/22:0)	negative	14.31	668.61938	[M+HCOO]-	Cer	MS/MS	0.0027	0.54
698	Cer(d18:0/22:1)	negative	13.78	666.60315	[M+HCOO]-	Cer	MS/MS	0.0045	0.49
753	Cer(d18:0/23:0)	negative	14.53	682.63507	[M+HCOO]-	Cer	MS/MS	0.0038	0.60
798	Cer(d18:0/24:0)	negative	14.76	696.65112	[M+HCOO]-	Cer	MS/MS	0.0041	0.53
794	Cer(d18:0/24:1)	negative	14.28	694.63568	[M+HCOO]-	Cer	MS/MS	0.0027	0.51
908	Cer(d18:0/26:1)	negative	14.72	722.66699	[M+HCOO]-	Cer	MS/MS	0.0027	0.40
747	Cer(d18:1/22:0)	negative	14.04	680.61829	[M-H]-	Cer	MS/MS	0.0027	0.56
1527	Cer(d18:1/22:1)	positive	13.39	620.59863	[M+H]+	Cer	MS/MS	0.0255	0.66
1614	Cer(d18:1/23:1)	positive	13.66	634.61414	[M+H]+	Cer	MS/MS	0.0242	0.66
836	Cer(d18:1/24:1)	negative	14.11	706.63373	[M+Hac-H]-	Cer	MS/MS	0.0202	0.66
894	Cer(d18:1/26:1)	negative	14.34	720.64746	[M+HCOO]-	Cer	MS/MS	0.0278	0.64
625	Cer(d38:1)	negative	13.19	638.56891	[M+HCOO]-	Cer	MS/MS	0.0071	0.55
915	Cer(d44:0)	negative	15.16	724.68146	[M+HCOO]-	Cer	MS/MS	0.0038	0.62
116	Linoleic acid	negative	2.50	279.23273	[M-H]-	FFA	STD	0.0387	0.65
365	PE(18:0/0:0)	negative	2.09	480.31	[M-H]-	PE	MS/MS	0.0316	0.66
986	PG(16:1/18:2)	negative	4.84	743.48297	[M-H]-	PG	MS/MS	0.0214	0.57

Remarks: MSMS = lipids that were confirmed with fragment pattern of database; STD = lipids that were confirmed with authentic standards. Abbreviations: Cer, ceramide; FFA, free fatty acid; PE, phosphatidylethanolamine; PG, phosphatidylglycerol.

Table S6. List of the mixture of 14 internal standards with corresponding statistical information in the lipidomics study using COVID-19 patients' and non-infected control subjects' plasma samples

Common Name	Formula	Detection mode	Adduct ion name	m/z	Retention time (min)	CV of plasma samples	CV of QC samples
Ceramide C17	C35H69NO3	Positive	[M+H-H2O]+	534.5250	9.88	21.97%	7.37%
Ceramide C17	C35H69NO3	Positive	[M+Na]+	574.5182	9.88	18.96%	6.35%
Cholesterol d7	C27H39D7O	Positive	[M-H2O+H]+	376.3960	5.89	21.07%	3.06%
DG (12:0/12:0/0:0)	C27H52O5	Positive	[M+Na]+	479.3728	4.60	10.16%	2.08%
DG (12:0/12:0/0:0)	C27H52O5	Positive	[M+NH4]+	474.4171	4.60	14.90%	1.24%
DG (18:1/2:0/0:0)	C23H42O5	Positive	[M+Na]+	421.2930	2.58	4.08%	1.49%
DG (18:1/2:0/0:0)	C23H42O5	Positive	[M+NH4]+	416.3379	2.58	9.20%	1.76%
LPC (17:0)	C25H52NO7P	Positive	[M+H]+	510.3575	1.59	20.99%	2.30%
LPE (17:1)	C22H44NO7P	Positive	[M+H]+	466.2947	1.29	13.51%	3.04%
PAF C-16-d4	C26H50D4NO7P	Positive	[M+H]+	528.3976	1.70	12.40%	2.34%
PC (12:0/13:0)	C33H66NO8P	Positive	[M+H]+	636.4587	3.31	12.53%	3.38%
PE (17:0/17:0)	C39H78NO8P	Positive	[M+H]+	720.5572	11.57	19.82%	7.12%
SM (17:0)	C40H81N2O6P	Positive	[M+H]+	717.5917	7.30	14.42%	3.65%
Sphingosine (d17:1)	C17H35NO2	Positive	[M+H]+	286.2730	1.14	14.11%	1.54%
TG (17:0/17:1/17:0)-d5	C54H97D5O6	Positive	[M+Na]+	874.7945	16.12	16.81%	5.31%
Ceramide (d18:1/17:0)	C35H69NO3	Negative	[M-H]-	550.5178	10.10	17.07%	6.57%
Arachidonic acid-d8	C20H24D8O2	Negative	[M-H]-	311.2837	2.04	23.36%	7.12%
LPE (17:1)	C22H44NO7P	Negative	[M-H]-	464.2787	1.27	13.13%	4.55%
PE (17:0/17:0)	C39H78NO8P	Negative	[M-H]-	718.5399	11.08	24.98%	4.98%
PG (17:0/17:0)	C40H79O10P	Negative	[M-H]-	749.5316	7.93	13.52%	3.58%

Abbreviations: DG, diacylglycerol; LPC, lysophosphatidylcholine; LPE, lysophosphatidylethanolamine; PC, phosphatidylcholine; PE, phosphatidylethanolamine; PG, phosphatidylglycerol; SM, sphingomyelin; TG, triacylglycerol.

Table S7. Lipids that were significantly different between COVID-19 patients and non-infected control subjects

Lipids id	Significant lipids	Detection mode	Retention time (min)	Accurate mass in detection mode	Adduct ion name	Lipid species	Identification information	p-Value in COVID-19 Patients vs Healthy volunteers	Fold change in COVID-19 Patients vs Healthy volunteers
112	Carnitine 10:2	positive	0.655	312.21759	[M]+	Car	MS/MS	0.0085	0.47
70	Carnitine 8:1	positive	0.646	286.20163	[M]+	Car	MS/MS	0.0002	0.50
138	Carnitine 11:1	positive	0.688	328.2485	[M]+	Car	MS/MS	0.0007	0.58
117	Carnitine 10:1	positive	0.686	314.23355	[M]+	Car	MS/MS	0.0432	0.60
252	O-oleoylcarnitine	positive	1.404	426.358	[M]+	Car	MS/MS	0.0001	1.62
859	(3'-sulfo)Galβ-Cer(d18:1/16:0)	negative	4.617	778.51257	[M-H]-	Cer	MS/MS	0.0001	0.66
1138	LacCer(d18:1/16:0)	negative	6.252	906.61615	[M+HCOO]-	Cer	MS/MS	0.0000	1.50
1203	LacCer(d18:1/24:1(15Z))	positive	13.099	1016.72742	[M+HCOO]-	Cer	MS/MS	0.0012	1.51
531	Cer(d18:1/24:1)	negative	13.829	646.61102	[M-H]-	Cer	MS/MS	0.0000	1.55
472	Cer(d18:1/18:0)	negative	11.693	610.54065	[M+HCOO]-	Cer	STD	0.0001	2.33
475	Cer(d18:0/18:0)	negative	12.844	612.55646	[M+HCOO]-	Cer	STD	0.0110	2.75
1501	SM(43:2)	positive	13.396	849.67871	[M+H]+	SM	MS/MS	0.0266	1.58
918	SM(36:0)	positive	9.353	733.62256	[M+H]+	SM	MS/MS	0.0027	1.77
1024	SM(38:2)	positive	14.17	757.62408	[M+Na]+	SM	MS/MS	0.0012	1.88
1009	SM(38:3)	positive	9.317	755.60309	[M+Na]+	SM	MS/MS	0.0013	2.10
226	PE(O-16:0/0:0)	negative	1.637	438.29785	[M-H]-	Ether-LPE	MS/MS	0.0350	0.42
251	PE(O-18:2/0:0)	negative	1.661	462.29794	[M-H]-	Ether-LPE	MS/MS	0.0337	0.50
1007	PC(O-35:4)	positive	6.948	754.57074	[M+H]+	Ether-PC	MS/MS	0.0000	0.33
1073	PC(O-18:0/18:5)	positive	6.654	766.5744	[M+H]+	Ether-PC	MS/MS	0.0014	0.35
951	PC(O-16:0/20:5)	negative	6.509	810.5658	[M-H]-	Ether-PC	MS/MS	0.0016	0.35
1193	PC(O-38:8)	positive	6.661	788.5556	[M+H]+	Ether-PC	MS/MS	0.0039	0.35
904	PC(O-33:2)	positive	7.272	730.5752	[M+H]+	Ether-PC	MS/MS	0.0000	0.40
1216	PC(O-16:0/22:6)	positive	6.779	792.59009	[M+H]+	Ether-PC	MS/MS	0.0033	0.45
967	PC(O-14:0/20:2)	positive	8.399	744.59192	[M+H]+	Ether-PC	MS/MS	0.0001	0.52

1016	PC(O-35:3)	positive	8.813	756.58789	[M+H]+	Ether-PC	MS/MS	0.0072	0.59
1055	PC(O-36:6)	positive	8.035	764.55658	[M+H]+	Ether-PC	MS/MS	0.0014	0.62
953	PC(O-34:3)	positive	8.036	742.57581	[M+H]+	Ether-PC	MS/MS	0.0006	0.65
1096	PC(O-36:3)	positive	8.573	770.60449	[M+H]+	Ether-PC	MS/MS	0.0011	0.66
987	PE(O-18:1/20:5)	positive	9.173	750.54388	[M+H]+	Ether-PE	MS/MS	0.0334	0.28
755	P(O-18:2/20:4)	negative	8.962	748.52795	[M-H]-	Ether-PE	MS/MS	0.0239	0.30
869	PE(P-16:0/20:5)	positive	6.875	722.51227	[M+H]+	Ether-PE	MS/MS	0.0048	0.32
683	PE(O-16:1/20:5)	negative	6.721	720.49683	[M-H]-	Ether-PE	MS/MS	0.0110	0.32
688	PE(O-16:1/20:4)	negative	7.033	722.51099	[M-H]-	Ether-PE	MS/MS	0.0001	0.41
787	PE(O-16:0/18:3)	positive	8.741	700.52716	[M+H]+	Ether-PE	MS/MS	0.0000	0.46
894	PE(P-18:0/18:2)	positive	11.631	728.55988	[M+H]+	Ether-PE	MS/MS	0.0000	0.48
887	PE(O-18:1/18:3)	positive	8.735	726.54431	[M+H]+	Ether-PE	MS/MS	0.0107	0.52
754	PE (P-16:0/22:5)	negative	7.875	748.52771	[M-H]-	Ether-PE	MS/MS	0.0043	0.56
775	PE(O-18:0/20:4)	negative	11.348	752.5592	[M-H]-	Ether-PE	MS/MS	0.0030	0.57
820	LNAPE(18:0/N-20:4)	negative	13.233	766.53961	[M-H]-	LNAPE	MS/MS	0.0000	3.30
1071	PC(32:2/0:0)	positive	8.383	766.573	[M+Na]+	LPC	MS/MS	0.0000	0.45
611	PC(26:0/0:0)	positive	6.126	636.49646	[M+H]+	LPC	MS/MS	0.0122	1.50
301	PE(20:5/0:0)	negative	0.971	498.26218	[M-H]-	LPE	MS/MS	0.0262	0.56
942	PC(34:4)	positive	6.109	740.55646	[M+H]+	PC	MS/MS	0.0000	0.28
1273	PC(16:1/22:6)	positive	4.727	804.55341	[M+H]+	PC	MS/MS	0.0309	0.51
1467	PC(40:2)	positive	13.388	842.66302	[M+H]+	PC	MS/MS	0.0003	0.54
1254	PC(37:3)	positive	8.35	798.60193	[M+H]+	PC	MS/MS	0.0192	0.62
1261	PC(19:0/18:2)	positive	10.923	800.61725	[M+H]+	PC	MS/MS	0.0011	0.63
1040	PC(18:0/18:2)	negative	9.335	844.60492	[M+CH3COO]-	PC	MS/MS	0.0216	1.73
935	PE(18:0/22:6)	negative	4.991	804.51801	[M-H]-	PE	MS/MS	0.0049	3.14
1081	PE(18:0/20:4)	positive	9.7	768.55402	[M+H]+	PE	MS/MS	0.0432	1.52
941	PE(36:4)	positive	7.307	740.52368	[M+H]+	PE	MS/MS	0.0028	1.78
1054	PE(38:6)	positive	6.117	764.52362	[M+H]+	PE	MS/MS	0.0385	1.94
672	PE(16:0/18:1)	negative	9.353	716.52338	[M-H]-	PE	MS/MS	0.0009	2.56

1630	PI(36:2)	positive	6.983	880.59125	[M+NH4]+	PI	MS/MS	0.0003	1.66

Remarks: MSMS = lipids that were confirmed with fragment pattern of database; STD = lipids that were confirmed with authentic standards; The 'O-' prefix is used to indicate the presence of an alkyl ether substituent, e.g., PC (O-16:0/0:0), whereas the 'P-' prefix is used for the 1Z-alkenyl ether (Plasmalogen) substituent, e.g., PE (P-16:0/16:0). Abbreviations: Cer, ceramide; EtherLPE, ether-linked lysophosphatidylethanolamine; EtherPC, ether-linked phosphatidylcholine; EtherPE, ether-linked phosphatidylethanolamine; LNAPE, n-acyl-lysophosphatidylethanolamine; LPC, lysophosphatidylcholine; PC, phosphatidylcholine; PE, phosphatidylethanolamine; PI, phosphatidylinositol; SM, sphingomyelin.
